# A New Approach for Developing “Implementation Plans” for Cognitive Stimulation Therapy (CST) in Low and Middle-Income Countries: Results From the CST-International Study

**DOI:** 10.3389/fpubh.2020.00342

**Published:** 2020-07-31

**Authors:** Charlotte R. Stoner, Mina Chandra, Elodie Bertrand, Bharath DU, Helen Durgante, Joanna Klaptocz, Murali Krishna, Monisha Lakshminarayanan, Sarah Mkenda, Daniel C. Mograbi, Martin Orrell, Stella-Maria Paddick, Sridhar Vaitheswaran, Aimee Spector

**Affiliations:** ^1^Centre for Chronic Illness and Ageing, Centre for Mental Health, Institute for Lifecourse Development, School of Human Sciences, University of Greenwich, London, United Kingdom; ^2^Atal Bihari Vajpayee Institute of Medical Sciences and Dr. Ram Manohar Lohia Hospital, Bangabandhu Sheikh Mujeeb Marg, New Delhi, India; ^3^Department of Psychology, Pontifical Catholic University of Rio de Janeiro, Rio de Janeiro, Brazil; ^4^Viveka Hospital, Mysore, India; ^5^Department of Psychology, Federal University of Rio Grande do Sul (UFRGS), Rio Grande do Sul, Brazil; ^6^Newcastle University Hospitals NHS Foundation Trust, Royal Vic Infirmary, Newcastle upon Tyne, United Kingdom; ^7^Dementia Care in Schizophrenia Research Foundation (DEMCARES in SCARF), Chennai, India; ^8^Occupational Therapy Department, Kilimanjaro Christian Medical University College, Moshi, Tanzania; ^9^Institute of Psychiatry, Psychology and Neuroscience, King's College London, London, United Kingdom; ^10^Institute of Mental Health, University of Nottingham, Nottingham, United Kingdom; ^11^Institute of Neuroscience, Newcastle University, Newcastle upon Tyne, United Kingdom; ^12^Research Department of Clinical, Educational and Health Psychology, University College London (UCL), London, United Kingdom

**Keywords:** translational research, implementation, cognition, developing countries, methodology, psychosocial, dementia

## Abstract

**Background:** Even with a strong evidence base, many healthcare interventions fail to be translated to clinical practice due to the absence of robust implementation strategies. For disorders such as Alzheimer's disease and other dementias, access to evidence-based interventions beyond research settings is of great importance. Cognitive Stimulation Therapy (CST) is a brief, group-based intervention, with consistent evidence of effectiveness.

**Methods:** An implementation focused, three-phase methodology was developed using extensive stakeholder engagement. The methods resulted in a standardized Implementation Plan for the successful translation of CST from research to practice. The methodology was developed using the Consolidated Framework for Implementation Research (CFIR) and refined in three countries that vary in levels of economic development and healthcare systems (Brazil, India and Tanzania).

**Results:** Five Implemention Plans for CST were produced. Each plan contained implementation strategies and action plans devised in conjunction with policy professionals, healthcare professionals, people with dementia and family carers, and an international team of researchers and clinicians.

**Conclusion:** This novel methodology can act as a template for implementation studies in diverse healthcare systems across the world. It is an effective means of devising socio-culturally informed Implementation Plans that account for economic realities, health equity and healthcare access.

## Introduction

Cognitive Stimulation Therapy (CST) is a brief, group-based, psychosocial intervention for people living with mild to moderate dementia (1). Manualised CST consists of 14 sessions of 45-min duration each, occurring twice a week for 7 weeks. Each session follows a theme (e.g., current affairs, word games, faces) and is designed to stimulate a range of cognitive abilities, whilst providing an optimal learning environment, and the social benefits of a group (2). The original CST programme referenced here was developed in the United Kingdom (UK), due to the limited efficacy of medication prescribed for dementia (3). It has a consistent evidence base for improving cognition and quality of life for people with dementia (4, 5). In particular, memory, comprehension of syntax and orientation appear to be most impact by CST, whilst impact on executive function, attention, and praxis has not been documented (6). CST has also been widely implemented in UK National Health Service (NHS) Memory Clinics (7) and is consistently recommended for people with dementia in the UK (8, 9). Whilst many programmes similar to CST have since been developed, the original UK version remains the most consistently evidenced (10–12).

As manualised CST was developed in the UK, some activities such as the use of a group song to open and close sessions, and reminiscence about childhood may be less appropriate in other contexts and cultures. To ensure that CST remains cross-culturally valid, a standardized methodology involving three distinct stages was developed, where adaptations are generated and reviewed in collaboration with stakeholders. Where this methodology has been adhered to, consistent evidence for the effectiveness of CST has emerged across countries (10). However, after adaptation, implementation is needed to maximize global uptake of evidence-based interventions.

Barriers to implementing evidence based interventions can occur at multiple levels of a health system including the patient level, provider or group level, organizational level and policy level (13) and there is a growing recognition of the importance of implementation research including evaluating what works, where and why across multiple contexts. The Consolidated Framework for Implementation Research (CFIR) (14) is a meta-theoretical amalgamation of 19 previous implementation models that can inform this process. It incorporates theories of innovation, organizational change, knowledge translation, uptake and dissemination and is designed to offer an overarching typology for constructs associated with implementation science, where constructs and domains can be used to guide and evaluate the implementation process. The CFIR has been used widely and can underpin diverse programmes, from weight management initiatives (15) to pharmacological interventions in addiction services (16).

There is no existing methodological framework for implementing healthcare interventions that also accounts for differing economic development of countries, healthcare systems, and complex implementation issues such as those contained within the CFIR. Thus, the aim of this paper is to present a newly developed, three-stage, implementation methodology that facilitates the successful implementation of evidence-based interventions like CST in diverse settings. Whilst evidenced for CST, the methodology can be generalized to other programmes and interventions across diverse contexts, thereby facilitating the translation of interventions from research to practice globally.

## Materials and Methods

Implementation Plans were developed separately for three countries (Brazil, India, and Tanzania) using the CFIR. The CFIR is a taxonomy of factors intrinsic to the implementation or “scaling up” of interventions from research to practice and has been used successfully in a number of interventions (15, 17). Consisting of five domains (intervention characteristics, outer setting, inner setting, characteristics of individuals and process) and 39 constructs, the CFIR can be used to guide and evaluate the implementation process across multiple and diverse healthcare systems. In particular, the continual engagement of stakeholders is advocated to explore the barriers to and facilitators of implementation and inform the implementation process at a provider, service user and organizational or political level (Table 1).

**Table 1:** Consolidated framework for implementation research (CFIR) domains and constructs.

**CFIR domain**	**Construct**
Intervention Characteristics	• Intervention source • Evidence strength and quality • Relative advantage • Adaptability • Trialability • Complexity • Design quality and packaging • Cost
Outer Setting	• Patient needs and resources • Cosmopolitanism • Peer pressure • External policies and incentives
Inner setting	• Structural characteristics • Networks and communications • Culture • Implementation climate
Characteristics of individuals	• Knowledge and beliefs about the intervention • Self-efficacy • Individual stage of change • Individual identification with organization • Other personal attributes
Process	• Planning • Engaging • Executing • Reflecting and evaluation

For the current project, the CFIR was used to develop a three-stage, mixed methodology employing both stakeholder engagement and the development of a quantitative scoring system. In summary, the methods consisted of: [1] exploration of barriers to and facilitators of CST implementation, [2] development of implementation activities to overcome each barrier or support each facilitator, and [3] development and monitoring of formal Implementation Plans. To demonstrate cross cultural validity, this methodology was used in three diverse countries with differing levels of economic development and differing healthcare systems: Brazil, India, and Tanzania. Brazil is an upper-middle income country, with both a public [Sistema Único de Saúde (SUS)] and private healthcare system (18). India is a lower-middle income country, with a public government healthcare system covering primary, secondary, and tertiary care. However, bottlenecks in accessing these services can occur, leading people to seek private care (19). Tanzania is a low-income country, where the health system is descentralised and is pyramidal in structure. Dispensaries serve local communities, followed by health centers, hospitals and larger referral hospitals (20).

The three countries chosen for this work were part of the CST-International research programme (21) and teams in each country had previously completed the formal adaptation of CST (22–24) using the established methodology (25). As this was a stakeholder project and not a formal research study, no identifying information was collected from stakeholders beyond their job title and no formal analysis was conducted.

### Phase 1: Exploration of the Barriers to and Facilitators of CST Implementation

#### Identifying Stakeholders

The Formative Method for Adapting Psychotherapy (FMAP) has previously been used to identify relevant stakeholders when adapting CST for different countries (25). This, combined with implementation theory, resulted in the identification of three groups of stakeholders from both public and private healthcare systems: Group [1] Decision makers or policy professionals who may commission or authorize the use of CST in services, group [2] healthcare professionals who may be expected to deliver CST as part of their regular duties, and group [3] those who may expect to receive CST and their supportive others.

#### Implementation Questions

All 39 constructs contained within the CFIR were examined and transformed into a series of questions addressing each of the five domains of implementation (see Supplementary Material). The questions were developed iteratively with researchers and clinicians from the UK, Brazil, India and Tanzania providing feedback and suggesting additional questions. Questions were designed to be all encompassing and cross-culturally valid. They could refer to differing awareness of dementia, healthcare systems and services including long-term care, private facilities and non-governmental organizations (NGOs). Further, questions were targeted according to stakeholder group and accounted for job role and responsibility, experience and expertise. A total of 39 questions were developed and a distinction was made between those considered essential to inform the implementation process and those deemed as supplementary. Thus, 15 questions (5 per group) were considered essential, with researchers in Brazil, India, and Tanzania required to ask them and 24 were classed as supplementary that could be asked if time allowed.

#### Stakeholder Meetings

Flexibility was needed when organizing stakeholder meetings and the format was amended to suit individual settings. However, all stakeholder meetings were required to be prefaced by introductory talks on both dementia and CST, ensuring equivalency of baseline knowledge. Further, all talks were tailored to ensure that the information presented was appropriate for the country or setting and the stakeholder group presented to. Following the talks, stakeholders were split into small groups, with one or two facilitators present. Facilitators asked each of the essential questions to the group, ensuring that all stakeholders were given an opportunity to express views. If there was time following thorough discussions of the essential questions, supplementary questions were put to stakeholders. Sessions were audio-recorded if attendees gave permission for this but as, this was not a formal research study, no formal qualitative analysis was undertaken.

### Phase 2: Development of Implementation Activities

#### Compilation of Barriers and Facilitators

Every barrier and facilitator identified across stakeholder meetings were synthesized into tables specific to each country. Each barrier or facilitator was then grouped according to the CFIR construct they referred to and were also grouped according to a more generic research theme. These tables were discussed by the primary team in each country and by the wider international team, who proposed implementation activities designed to overcome barriers or reinforce facilitators identified. Researchers were asked to focus on tangible and realistic activities that could be achieved as part of the CST-International research programme (2018–2021). Activities proposed by the primary team in each country were subsequently discussed and agreed upon in teleconferences with the UK based team. All agreed upon activities were added to the table, next to the corresponding barrier or facilitator.

#### Reaching Consensus on Implementation Activities Proposed

**Figure 1:**
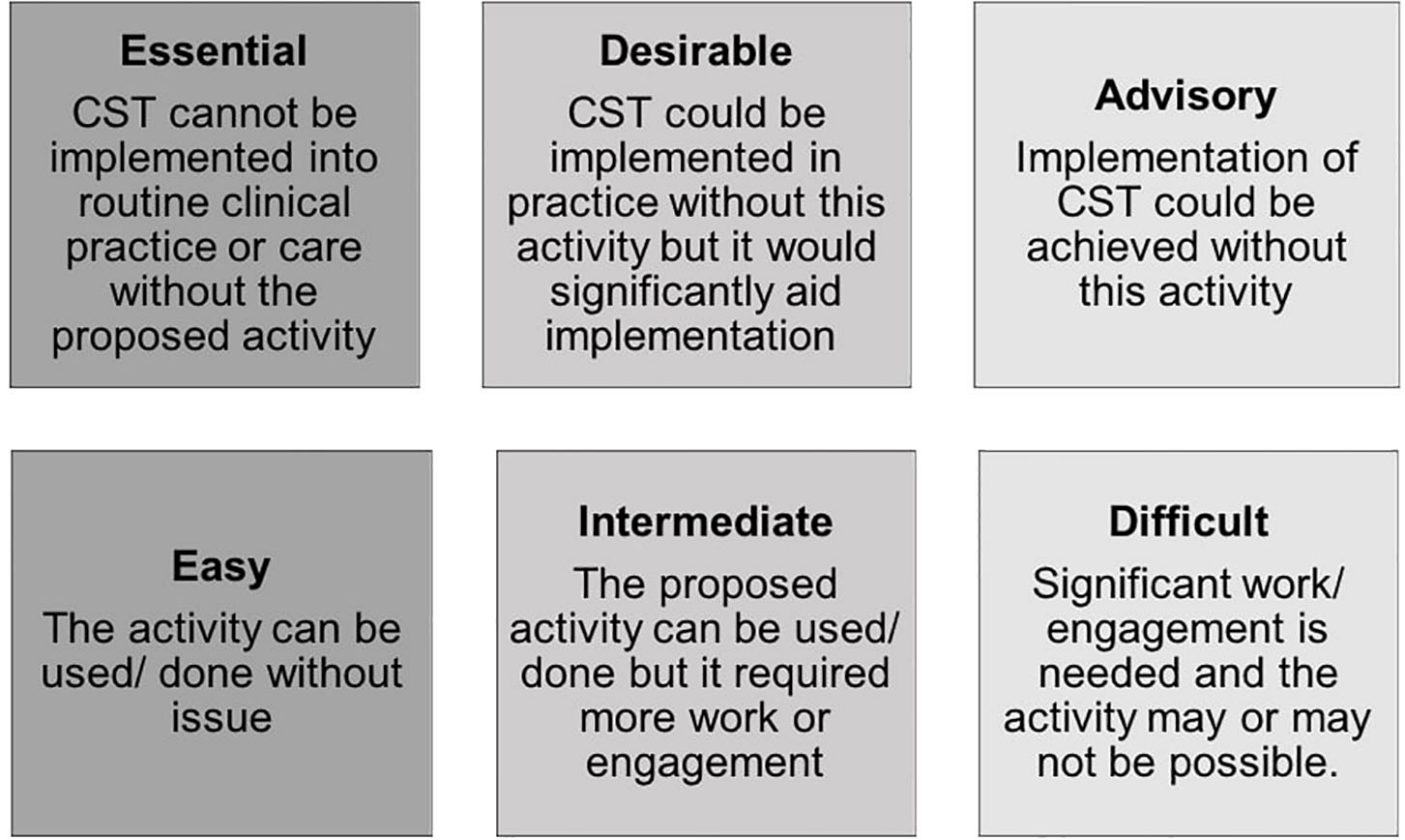
Rating matrix for implementation activities.

As numerous barriers and facilitators were identified, the corresponding number of implementation activities was also high. Recognizing that it might not be feasible to use all activities proposed, a system was devised to ensure that activities considered essential to the successful implementation of CST were prioritized. Further, the team recognized that implementation activities were of ranging difficulty practically. As such, a secondary rating system was added to ensure that activities that were of relative ease were also prioritized. This resulted in a two-by-three matrix system (26), where both the perceived importance and ease of use for each implementation activity proposed could be captured. It was also important to ensure that stakeholders were re-engaged with to determine the importance and difficulty of each proposed activity. Thus, the completed table with barriers, facilitators and proposed activities were circulated firstly to each research team and then to all other stakeholders for feedback. Both the research team and stakeholders were asked to provide two ratings for each activity according to the two-by-three matrix (Figure 1).

It was stipulated that all stakeholders should be invited to provide ratings, in order to maximize the response rate. To analyse responses, a scoring system was used for the matrix where scores ranged from one (activity was both advisory and difficult) to 9 (activity was both essential and easy). The mode(s) for each activity was then calculated and results discussed during a consensus meeting in each country. Consensus was reached when members of the primary team in each site formally agreed upon which activities would be undertaken, prioritizing those with the highest modes judged most feasible. The teams did this by further splitting activities into those that were “essential” for successful implementation and those that the team would “further consider” if time and resources allowed.

### Phase 3: Writing and Monitoring the Implementation Plan

The agreed upon implementation activities were formalized in a research document called an “Implementation Plan.” Whilst plans could be written in the format or style most suited to each context, it was stipulated that all teams were required to continue using the CFIR and that each Implementation Plan should include:

1. A written summary of the barriers and facilitator tables, detailing which implementation activities were to be undertaken and the CFIR construct they referred to.
2. Justification for the number of activities that the team agreed to undertake, with reference to available time and resources.
3. Justification for decisions not to undertake an activity that had a high mode (8 = essential and intermediate or 9 = essential and easy), where applicable.
4. “Action plans” for the site where each activity to be undertaken was assigned to a member of staff and given a due date for when the action should be completed by.
5. A local barrier and facilitator checklist to be used by newly trained facilitators before commencing a CST group addressing logistical issues commonly faced when beginning CST.

After the Implementation Plan had been written and agreed upon by the local and international teams, it was routinely monitored by staff to ensure that actions were undertaken. Staff were encouraged to keep a record of completed actions and a research diary detailing which activities were successful and which were less so.

An overview of the methodology and process, with example results can be found in Table 2. More detail on results from each phase can be found in subsequent sections.

**Table 2:** Methodology overview and example results.

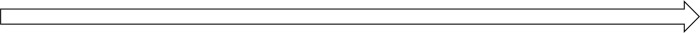					
	**Phase 1**		**Phase 2**		**Phase 3**
**Country**	**Question asked *(CFIR construct)***	**Barrier/Facilitator identified**	**Implementation activity developed**	**Rating of activity by stakeholders**	**Refinement and inclusion in Implementation Plan**
Brazil	What training/support will staff need to implement CST e.g., time off regular duties/travel for training? *(implementation climate)*	Healthcare professionals, who might be expected to run CST work long hours, with competing demands. This means they may not have time to facilitate CST groups.	Venues offering CST groups will be required to guarantee staff are granted protected time for running groups and attending training	Essential and of Intermediate difficulty	Essential: CST facilitators will need protected time allocated for CST (training and running sessions). The research team should inform site management that protected time for staff is a requirement of the project when approaching/recruiting sites and ensure that site managers agree to this.
India	What organizations/charities/government bodies are available to you to help support implementation? *(cosmopolitanism)*	There is a lack of awareness of CST as a treatment option. Networking with other organizations may improve awareness.	Research team to advertise CST through media and form a network of organizations that are interested in including CST in their service	Essential and of Intermediate difficulty	Essential: Researchers to advertise CST and create a network of organizations that show interest in CST who are regularly updated with local resources and use of the intervention
Tanzania	What are the known barriers people encounter when accessing services? *(patient needs and resources)*	Some older adults have visual impairments but no access to eye care. Visual impairments that cannot be compensated for will limit engagement with CST.	Screening for CST suitability should include a brief eye test	Desirable and of Intermediate difficulty	Further Consider: As part of screening procedures, participants can be referred to an ophthalmologist to address any visual impairments prior to commencing CST.

## Results

### Phase 1: Exploration of the Barriers to and Facilitators of CST Implementation

#### Brazil

Four meetings took place between November 2018 and January 2020 in Rio de Janeiro and São Paulo involving 50 stakeholders (Group 1 = 15, Group 2 = 20, and Group 3 = 15). In Rio de Janeiro all three groups attended one session where they listened to introductory talks to dementia and CST by the research team before dividing into smaller groups, according to their designation (Group 1, 2, or 3) for the discussion. In São Paulo, three meetings where facilitated by the research team, with each stakeholder group attending introductory talks and taking part in small group discussions. Group 1 stakeholders included administrative directors of public health services and NGOs, primary care coordinators, private clinic administrators and private clinic administrators. Group 2 stakeholders included psychologists, nurses, speech therapists, social workers and occupational therapists. Group 3 consisted of people with dementia, their family members or supportive others and other members of the general public with experience of or an interest in dementia. In both locations, common barriers identified by Group 1 included a lack of awareness regarding treatment policies or guidelines for dementia or how policies were applied practically in the varied healthcare services. Group 2 stakeholders noted there could be high staff burden, with healthcare professionals lacking capacity to deliver what was perceived as an extra service alongside their usual duties. Group 3 stakeholders discussed a lack of support from the public sector and high levels of stigma.

#### India

Across three sites (Mysore, Chennai and New Delhi) a total of 77 stakeholders took part in discussions (Group 1 = 22, Group 2 = 31, and Group 3 = 24). Group 1 stakeholders included Government officers in charge of Ministry of Health programmes, representatives from the Alzheimer's and Related Disorders Society of India (ARDSI) and decision makers NGOs. Group 2 representatives included psychiatric nurses, research assistants, consultant psychiatrists, neurologists, geriatricians, neurology, and psychiatry residents and psychologists. For Group 3, people with dementia, family caregivers and community leaders responded. In all sites, Group 1 stakeholders were interviewed individually, due to their limited availability. In Chennai, the decision was made to speak to nurses and nursing assistants separately from medical doctors to ensure that nurses were comfortable giving a view. Group 1 responders across sites noted that there was often a lack of national funding available for research and services for people with dementia and there was often a lack of communication between key policy professionals and professionals. Group 2 noted that stigma and the cost of services could impact attendance for dementia services and that psychosocial interventions were perceived as less valuable than medical interventions. Caregivers in Group 3, who had taken part in previous psychosocial interventions in Chennai, suggested that they viewed these interventions as beneficial, improving communication for the person with dementia. They also noted some logistical barriers including transportation difficulties and scheduling conflicts as caregivers had to take time off from employment to accompany persons with dementia to attend sessions.

#### Tanzania

Two meetings took place in Arusha and Moshi in October 2018 involving 49 stakeholders (Group 1 = 5, Group 2 = 33, and Group 3 = 11). Group 1 stakeholders included directors of services at local hospitals, regional mental health coordinators and heads of departments in a local hospital. Group 2 were mostly nurses, medical doctors, counselors and psychologists. Group 3 consisted of five people with dementia and six carers or supportive others. The people with dementia had previously participated in a pilot study of CST. Barriers identified in Tanzania included a lack of awareness of dementia as a disease, under-developed transport networks limiting the degree to which people with dementia could travel to a group and, similar to Brazil, high staff burden. However, carers and people with dementia in Group 3 described the benefits of meeting with their peers and the sharing of news across villages and towns. There were also more practical barriers identified in Tanzania associated with running CST groups. First, some sessions required the use of electronic equipment and electricity could not always be relied upon in more rural areas. Second, a small but significant proportion of older adults spoke only Chaga languages, thus creating a language barrier with some of their fellow attendees and Swahili speaking healthcare professionals. Third, meeting spaces could be difficult to source, with previous groups taking place in churches or school. Both were described as problematic with the former potentially leading to exclusion and the latter to embarrassment.

### Phase 2: Development of Implementation Activities

#### Compilation of Barriers and Facilitators

##### Brazil

A total of 58 barriers and facilitators were identified across both sites within CFIR categories of: inner setting (implementation readiness, networks and communications, structural characteristics), outer setting (patient needs/resources, external policies, and incentives), intervention characteristics (cost, relative advantage, evidence strength and quality) and characteristics of individuals (knowledge and beliefs about the intervention). To overcome each barrier or support each facilitator, 41 implementation activities were proposed, with a minimum of one activity proposed for each area of the CFIR but activities proposed could address multiple barriers or facilitators.

##### India

As the India sites were diverse, research teams in each site wrote and compiled their own barrier and facilitator documents. In Chennai, a total of 133 barriers and facilitators were identified, and 49 implementation activities proposed. In Mysore, 26 barriers and facilitators were identified, and e 62 identified activities to support implementation were proposed. Finally, in New Delhi, a total of 25 barriers and facilitators were identified, and 62 activities proposed (country total: 184 barriers/facilitators and 173 activities proposed). Activities fell under the CFIR domains of inner setting (structural characteristics, implementation climate, networks, and communications), outer setting (peer pressure, patient needs/resources, external policies and incentives, cosmopolitanism), intervention characteristics (complexity, cost, adaptability, design quality and packaging, evidence strength and quality, relative advantage) and characteristics of individuals (knowledge/beliefs about the intervention, motivation, values, self-efficacy).

##### Tanzania

A total of 55 barriers and facilitators were documented in Arusha and Moshi, within the CFIR categories of: inner setting (networks and communications, structural characteristics, culture), outer setting (patient needs/resources), intervention characteristics (cost, relative advantage, design quality, and packaging), and characteristics of individuals (knowledge/beliefs about the intervention). A total of 41 implementation activities were proposed to overcome all barriers and support all facilitators. For Tanzania, the research team discussed whether it was more feasible for CST facilitators to transport people with dementia who lived in very rural setting or whether it was feasible for people with dementia and carers to arrange their own transport. Ultimately a decision could not be made and so both activities were proposed, with the decision postponed until stakeholders could express a preference.

#### Reaching Consensus on Implementation Activities Proposed

##### Brazil

Thirty-three stakeholders from Phase 1 rated the 41 implementation activities according to their perception of how essential each activity was for successful implementation and how easy each were to do. Responders consisted of five members of the CST-International research team (two psychologists, one psychiatrist, one PhD candidate, and one research assistant) and 16 potential CST facilitators (Group 2), for whom professions included psychologists, nurses, physicians, gerontologists, speech therapists, recreation workers, and social workers. Six decision makers from the private and public sectors responded on behalf of Group 1 (local policy or decision makers). For Group 3 (people with dementia, caregivers and other interested parties), six caregivers responded.

The most common individual rating for activities was 8 (Essential and Intermediate), however, there was a large amount of variance in responses. For example, the implementation activity “CST training should be delivered where CST sessions are going to be offered” was rated as advisory and intermediate (2) by two responders and essential and easy (11) by two responders. All activities were discussed at a local team meeting, with reference to how many could be implemented and the prioritization of those activities that were rated as both essential for implementation and easy to use. It was decided that activities rated as essential and easy/intermediate to use would always be categorized as “essential” and activities with lower modes would be categorized as “further consider.” A total of 22 activities were considered essential for implementation success and nine were designated as further consider if time and resources allowed.

##### India

In Chennai, 12 stakeholders rated how essential and easy the identified activities to support implementation were. Responders consisted of four members of the CST-International research team (three consultant psychiatrists and one psychologist). Two decision makers from an NGO responded on behalf of Group 1. For Group 2, two potential CST facilitators (psychologists) and two psychiatrists responded. For Group 3, two caregivers responded.

The most common individual ratings for activities were 9 (Essential and Easy) and 8 (Essential and Intermediate). All activities were discussed at a local research team meeting, where it was decided activities rated as essential and easy/intermediate to implement would always be categorized as “essential” (40 activities). Some activities rated six (Desirable and Intermediate) or below were deemed too resource intensive. For example, under “Inner Setting” a mechanism that required “researchers to identify homes willing to deliver CST and pitch CST to them” was considered too resource intensive, involving creating an exhaustive list of care homes in Chennai. Six activities were further considered by the team.

In Mysore, 13 stakeholders rated the implementation activities. Responders consisted of four members of the CST-International research team (three psychiatrists and one speech pathologist) and three potential CST facilitators (Group 2) that included two psychologists and one psychiatric social worker. Three stakeholders responded on behalf of Group 1 and included a head of Department in Psychiatry in a State-run Medical College, a District Mental Health Officer and a District Psychiatrist. For Group 3, two caregivers and one person with dementia responded. All activities were discussed at a local team meeting, where it was decided that activities rated as essential and easy/intermediate (9/8) to implement would always be categorized as “essential” and activities with lower modes would be categorized as “further consider”. This resulted in 16 “essential” activities and eight “further consider” activities.

For New Delhi, all identified stakeholders from Phase 1 rated the implementation activities (Group 1 = 10, Group 2 = 10, Group 3 = 8). All domain areas considered essential were shortlisted as mandatory for CST implementation (45 “essential” activities) while the consensus view was that domain areas considered advisory and desirable should be left to the discretion of the team depending on available resources and logistics. These nine activities were designated as “further consider.”

##### Tanzania

16 stakeholders rated implementation activities for Arusha and Moshi. These consisted of four members of the CST-International team (two geriatricians, one registrar, and one senior research associate) and eight potential CST facilitators (Group 2), for whom professions included psychologists, medical officers and occupational therapists. A decision maker at a large university hospital responded on behalf of Group 1 (local policy or decision makers). For Group 3 (people with dementia, caregivers and other interested parties), two caregivers and one person with dementia responded.

The most common individual rating for activities was 6 (Desirable and Intermediate), however, there was a large amount of variance in responses. For example, the activity “psychoeducation should include information on the underlying pathology of neurodegenerative diseases” was rated as advisory and difficult (1) by two responders and essential and easy (11) by two responders. All activities were discussed at a local team meeting, with reference to how many could be implemented and the prioritization of those activities that were rated as both essential for implementation and easy to use. It was decided that activities rated as essential and easy/intermediate to implement would always be categorized as “essential” (15 activities) and activities with lower modes would be categorized as “further consider” (seven activities).

### Phase 3: Writing and Monitoring the Implementation Plan

#### Brazil

The São Paulo and Rio de Janeiro sites were considered similar enough to allow for one Brazil Implementation Plan, with specific actions attributed to sites where appropriate. A draft implementation plan was circulated, and CST-Investigators were invited to comment and refine the plan. The fifth and final iteration of this plan was reviewed and approved by all members of the team. The 22 essential activities recorded in the Brazil Implementation Plan fell under CFIR domains of: knowledge/beliefs about the intervention, relative advantage, costs (direct and indirect), networks and communications, external policy and incentives and structural characteristics. The nine further consider activities fell under the CFIR categories of: patient needs and resources, adaptability, structural characteristics and individual stage of change. Ten members of the research across sites were assigned action plans consisting of their essential and further consider actions. An example of a Rio de Janeiro based researcher's action plan is given in Table 3. The Brazil local barriers and facilitators checklist contained four subsections: participants, facilities, travel and timing and materials. Example items included “Is group to be held in a neutral setting (e.g., it is not in a church)?,” “can the building be reached by public transport?,” and “have you got all the electronic devices needed for sessions (e.g., mobile phone with songs preloaded)?

**Table 3:** Example action plan for Rio de Janeiro based researcher.

**CFIR construct**	**Action point**	**Due by**	**Completed on**
**ESSENTIAL**			
Patient needs and resources	CST-Investigators should approach leaders in community settings as possible sites offering CST	31.07.2020	
Patient needs and resources	CST-Investigators should approach managers of hospital outpatient clinics as possible sites offering CST	31.07.2020	
Relative advantage	CST-Investigators will explain the evidence-based benefits and the cost-effectiveness analysis indicating CST is a cheaper alternative to sites managers when approaching/recruiting sites	31.07.2020	
External policy and incentives	Contact with government stakeholders to discuss the possibility of implement CST at a policy level	31.08.2021	
**FURTHER CONSIDER**			
Adaptability	Adapt CST material for illiterate people, people with disabilities and people with severe dementia	31.08.2021	

#### India

The India sites were considered too diverse for one Implementation Plan and, therefore, three local level plans were first developed. Each plan was developed iteratively with feedback from other Indian sites and from the international team. In Chennai, the 39 essential activities fell under CFIR constructs of: structural characteristics, implementation climate, external policies and incentives, patient needs and resources, cosmopolitanism, knowledge/beliefs about the intervention, other attributes, costs (direct and indirect), relative advantage, complexity, and evidence strength. The six further consider activities fell under: structural characteristics, external policies and incentives, patient needs and resources, costs (direct and indirect), and complexity.

In Mysore, the 16 essential activities fell under the CFIR constructs of: patient needs and resources, knowledge/beliefs about the intervention, costs (direct and indirect), design quality and packaging, network and communications, structural characteristics, adaptability, complexity, and implementation climate. Further consider activities (10) fell under: patient needs and resources, knowledge/beliefs about the intervention, costs (direct and indirect), structural characteristics, adaptability, complexity, and implementation climate.

In New Delhi, 55 activities were considered essential and seven were further considered. Essential activities fell under the CFIR domains of interventions characteristics, inner setting and outer setting. Examples specific essential activities under the domain of intervention characteristics included highlighting the advantages of group therapy during educational initiatives for carers and the use of culturally heterogeneous CST groups for participants.

Across all plans, seven researchers were assigned action plans and an example of a Mysore based researcher action plan is given in Table 4. Local barriers and facilitator checklists were largely similar across sites and all contained the same subsections as the Brazil checklist (participants, facilities, travel and timing and materials). Example items from the Mysore checklist included, “Are there enough chairs?,” and “Have you agreed which times and dates CST sessions will be with carers?”

**Table 4:** Example action plan for Mysore based researcher.

**CFIR construct**	**Action point**	**Due by**	**Completed on**
**ESSENTIAL**			
Knowledge/beliefs about intervention	Local advertising about CST in collaboration with NGOs and local media. Contact details should be provided if people want to get more information	1/6/2020	
Knowledge/beliefs about intervention	Adding information about global effects of CST, advantages of group therapy to the Dementia Awareness Course (DAC) delivered to carers	Completed	
Design quality and packaging	All CST facilitators should be given a local checklist and asked to complete this checklist prior to running their first CST group	Completed	
Network and communication	Supervision of CST Facilitators and online mentoring	1/6/2020 On-going	
Complexity	Include information about delayed results/prolonged duration of CST (7 weeks, 14 days) in Dementia Awareness Course (DAC) and advantages of group therapy, respite for caregivers, global effects of CST.	Completed	
**FURTHER CONSIDER**			
Structural characteristics	Deliver CST facilitator training course to potential paramedical staff and hiring these personnel exclusively for CST delivery	1/6/2021	

#### Tanzania

As in Brazil, it was decided that the Moshi and Arusha areas were similar enough to necessitate the use of one Implementation Plan. The plan was finalized over three iterations and the 15 essential actions fell under the CFIR categories of: patient needs and resources, knowledge/beliefs about the intervention, costs (direct/indirect), design quality and packaging, network and communications, structural characteristics, and culture. The seven further consider activities fell under the CFIR constructs of patient needs and resources, knowledge/beliefs about the intervention, costs (direct and indirect) and structural characteristics. Ten members of the team were assigned action plans, an example of which is given in Table 5. The local barriers and facilitators checklist contained items such as “Will there be access to drinking water?,” “Have you checked when the local market day is?” and “Is there a contingency plan for sessions where you will need electricity?”

**Table 5:** Example action plan for Tanzania researcher.

**CFIR Construct**	**Action Point**	**Due by**	**Completed on**
**ESSENTIAL**			
Knowledge/beliefs about intervention	Oversee the contacting of higher education institutes (focusing on nursing, occupational therapy, psychology) to discuss including CST in taught programmes, with reference to resources needed by institute.	30.08.2020	

## Discussion

Using an innovative methodology developed using the CFIR and extensive stakeholder engagement, five Implemention Plans for CST were produced for three countries. The systematic development of methods in implementation research in LMICs has previously been suggested as an important means of facilitating cross-culturally valid research in this setting (27). The methods described here represent a first step in achieving this goal.

Each plan contained implementation strategies devised in conjunction with policy professionals, healthcare professionals, people with dementia and family carers, and an international team of researchers and clinicians. The use of this methodology resulted in unique plans suitable for diverse contexts including NGOs, public health services and private clinics in countries with varying levels of economic development or infrastructure. For example, the Brazil Implementation Plan contained strategies to implement CST in both private clinics and in the public system. In India, creating networks and collaborations between both NGOs and government healthcare facilities to facilitate information sharing was prioritized. In Tanzania, increasing awareness of dementia and treatment options amongst all stakeholders was a necessary step for successful implementation. The individualistic nature of the developed plans illustrates the flexibility of this methodology when applied to diverse contexts.

Barriers and facilitators documented here were consistent with those previously identified. A shortage of qualified professionals was identified here as a barrier to delivering CST and this shortage across services has also been suggested as negatively impacting on how a service user evaluates the quality of a healthcare service (28). In Brazil, it has been suggested that CST could be viewed as additional work for both caregivers and healthcare professionals (24). This is consistent with the barrier of healthcare professionals having competing demands on their time documented here. In India, the role of NGOs was identified here as an important facilitator for CST, creating networks across other NGOs and to government healthcare facilities. NGOs such as the 10/66 Research Group, have been identified as playing an important role in the facilitation of knowledge sharing both within India and internationally (29).

Whilst there is consistent evidence for the effectiveness of CST, less is known about its implementation to routine clinical care. To our knowledge, this is the first paper that provides a practical, evidence-based methodology for planning the systematic implementation of CST for people with dementia in diverse contexts. The resulting “Implementation Plans” developed as a result of using this methodology can be used to determine how best to implement CST. This moves the body of research concerning CST forward, from successful adaptation of the CST manual to effective implementation in varying countries and healthcare systems.

The CFIR provides a pragmatic structure for identifying, organizing and exploring constructs associated with implementation for healthcare interventions. In contrast to other studies where individual domains have been selected (30), all domains of the CFIR were examined and included in the current methods. This enabled an in-depth and holisitic exploration of the barriers to and facilitators of implementation in three diverse countries. Whilst these methods specifically address implementation issues for CST in different contexts, the methodology used can be generalized to other interventions in diverse countries.

### Methodological Problems and Limitations

The methodology here was developed drawing on implementation science. However, as it was novel, there were some difficulties observed during its use as part of the CST-International trial. Examples of methodological problems presented here can act as further guidance or considerations for future use of the methodology. During Stage 1 in the Rio de Janeiro site, it was noted that during a small stakeholder discussion group of potential CST facilitators, the group was being led by a member of staff who was the hierarchical superior of the stakeholders. This may have influenced the answers given, however, no other groups were led by managers, limiting the effect of this.

During Phase 2, a large amount of variance was sometimes observed for activities in Tanzania, with the implementation activity “dementia awareness course for family carers should contain information on stigma associated with dementia” rated as essential/easy and advisory/difficult. It was not clear why there was a large variance and formal qualitative interviews alongside may have helped.

## Future Research

Whilst not included here, a further Implementation Plan for Trissur using the same methodology is in development. Once this plan is completed site leads will hold a consensus meeting to synthesize the local plans and create one National Implementation Plan for India. This plan will include information that is deemed relevant across sites and will ensure that the activities proposed target the full range of institutions involved with dementia care or treatment such as NGOs, government hospitals and charities.

This methodology is proposed as a “gold standard” for the implementation of CST and further evidence will be evaluated in future work planned in China. It should also be tested in more diverse settings to ensure the methodology can be used effectively in both LMICs and high-income countries (HICs). The methodology will also be disseminated via the International CST Centre, hosted by University College London (UCL) to ensure implementation of CST follows best practice for both the adaptation, and the implementation stage. The effectiveness of the strategies in Brazil, India and Tanzania will be evaluated as part of the CST-International body of work (21).

## Conclusion

A practical, evidence-based methodology for the successful implementation of CST in diverse countries and healthcare systems was developed using implementation science frameworks. The methodology has been successfully used to create “Implementation Plans” for CST in Brazil, India, and Tanzania with further work in other countries planned. This methodology is the first of its kind and could be used as a template for the implementation of other non-pharmacological interventions for people with dementia, particularly in LMICs.

## Data Availability Statement

The original contributions presented in the study are included in the article/Supplementary Material, further inquiries can be directed to the corresponding author/s.

## Author Contributions

The methodology described here was developed primarily by CS and AS, with support from all other co-authors. The manuscript was written by CS, with authors MC, BD, MK, ML, and SV contributing to information from India. EB, HD, and DM contributing to Brazil information and JK, SM, and S-MP contributing to Tanzania information. MO provided methodological advice and commented on drafts of the manuscript. All authors contributed to the article and approved the submitted version.

## Conflict of Interest

AS offers Cognitive Stimulation Therapy (CST) training courses on a consultancy basis. AS, MO, and CS are co-authors of the CST manuals. Royalties for these manuals are received by University College London. The remaining authors declare that the research was conducted in the absence of any commercial or financial relationships that could be construed as a potential conflict of interest.

## References

[1] Spector A Thorgrimsen L Woods B Royan L Davies S Butterworth M. Efficacy of an evidence-based cognitive stimulation therapy programme for people with dementia: randomised controlled trial. Br J Psychiatry. (2003) 183:248–54. 10.1192/bjp.183.3.248 12948999

[2] Spector A Orrell M Davies S Woods B Can reality orientation be rehabilitated? Development and piloting of an evidence-based programme of cognition-based therapies for people with dementia. Neuropsychol Rehab. (2001) 11:377–97. 10.1080/09602010143000068

[3] Orrell M Woods B Editorial Comment. Tacrine and psychological therapies in dementia — no contest? Int J Geriatr Psych. (1996) 11:189–92. 10.1002/(SICI)1099-1166(199603)11:3&lt;189::AID-GPS312&gt;3.0.CO;2-K

[4] Woods B Aguirre E Spector AE Orrell M. Cognitive stimulation to improve cognitive functioning in people with dementia. Cochr Database Syst Rev. (2012) CD005562. 10.1002/14651858.CD005562.pub2 22336813

[5] Aguirre E Hoare Z Streater A Spector A Woods B Hoe J. Cognitive stimulation therapy (CST) for people with dementia—who benefits most? Geriat Psych. (2013) 28:284–90. 10.1002/gps.3823 22573599

[6] Hall L Orrell M Stott J Spector A. Cognitive stimulation therapy (CST): neuropsychological mechanisms of change. Int Psych. (2013) 25:479–89. 10.1017/S1041610212001822 23146408

[7] Royal College of Psychiatrists Memory Services National Accreditation Programme Fourth National Report: 2015–2016. London: Royal College of Psychiatrists' Centre for Quality Improvement (2016).

[8] National Institute for Health and Care Excellence (NICE) Dementia: Supporting People With Dementia and Their Careers in Health and Social Care. London: NICE (2006).

[9] National Institute for Health and Care Excellence (NICE) Dementia: Assessment, Management and Support for People Living With Dementia and Their Carers. London, NICE (2018). 30011160

[10] Lobbia A Carbone E Faggian S Gardini S Piras F Spector A The efficacy of cognitive stimulation therapy (CST) for people with mild-to-moderate dementia: a review. Europ Psychol. (2018) 11:434–41. 10.1590/1980-57642016dn11-040014

[11] Gibbor L Yates L Volkmer A Spector A. Cognitive stimulation therapy (CST) for dementia: a systematic review of qualitative research. Aging Mental Health. (2020) 1–11. 10.1080/13607863.2020.1746741. [Epub ahead of print]. 32252561

[12] International Cognitive Stimulation Therapy (CST) Centre Cognitive Stimulation Therapy (CST) By Country. Available online at: from ucl.ac.uk/international-cognitive-stimulation-therapy (2020).

[13] Ferlie EB Shortell SM. Improving the quality of health care in the United Kingdom and the United States: a framework for change. Milbank Q. (2001) 79:281–315. 10.1111/1468-0009.00206 11439467 PMC2751188

[14] Damschroder LJ Aron DC Keith RE Kirsh SR Alexander JA Lowery JC. Fostering implementation of health services research findings into practice: a consolidated framework for advancing implementation science. BMC Implem Sci. (2009) 4:50. 10.1186/1748-5908-4-50 19664226 PMC2736161

[15] Damschroder LJ Goodrich DE Robinson CH Fletcher CE Lowery JC. A systematic exploration of differences in contextual factors related to implementing the MOVE! weight management program in VA: A mixed methods study. BMC Health Serv Res. (2011) 11:248. 10.1186/1472-6963-11-248 21961925 PMC3206421

[16] Green CA McCarty D Mertens J Lynch F L Hilde A. (2014). A qualitative study of the adoption of buprenorphine for opioid addiction treatment. J Subst Abuse Treat. (2014) 46:390–401. 10.1016/j.jsat.2013.09.002 24268947 PMC3897203

[17] (2009) 4 Keith RE Crosson JC O'Malley AS Cromp D Taylor EF. Using the Consolidated Framework for Implementation Research (CFIR) to produce actionable findings: a rapid-cycle evaluation approach to improving implementation. Implem Sci. (2017) 12:15. 10.1186/s13012-017-0550-7 28187747 PMC5303301

[18] Massuda A Hone T Leles FAG de Castro MC Atun R. The Brazilian health system at crossroads: progress, crisis and resilience. BMJ Global Health. (2018) 3:e000829. 10.1136/bmjgh-2018-000829 29997906 PMC6035510

[19] Prinja S Aggarwal AK Kumar R Kanavos P. User charges in health care: evidence of effect on service utilization & equity from north India. Indian J Med Res. (2012) 136:868−76. 23287137 PMC3573611

[20] Manzi F Schellenberg JA Hutton G Wyss K Mbuya C Shirima K. Human resources for health care delivery in Tanzania: a multifaceted problem. BMC Hum Resour Health. (2012) 10:3–3. 10.1186/1478-4491-10-3 22357353 PMC3311084

[21] Spector A Stoner CR Chandra M Vaitheswaran S Du B Comas-Herrera A. Mixed methods implementation research of cognitive stimulation therapy (CST) for dementia in low and middle-income countries: study protocol for Brazil, India and Tanzania (CST-International). BMJ Open. (2019) 9:e030933. 10.1136/bmjopen-2019-030933 31434784 PMC6707660

[22] Mkenda S Olakehinde O Mbowe G Siwoku A Kisoli A Paddick S. Cognitive stimulation therapy as a low-resource intervention for dementia in sub-Saharan Africa (CST-SSA): adaptation for rural Tanzania and Nigeria. Dementia. (2016) 17:515–30. 10.1177/1471301216649272 27328694

[23] Raghuraman S Lakshminarayanan M Vaitheswaran S Rangaswamy T. Cognitive stimulation therapy for dementia: pilot studies of acceptability and feasibility of cultural adaptation for India. Am J Geriatr Psych. (2017) 25:1029–32. 10.1016/j.jagp.2017.04.014 28545833

[24] Bertrand E Naylor R Laks J Marinho V Spector A Mograbi DC. Cognitive stimulation therapy for brazilian people with dementia: examination of implementation' issues and cultural adaptation. Aging Mental Health. (2018) 23:1–5. 10.1080/13607863.2018.1488944 30444133

[25] Aguirre E Spector A Orrell M. Guidelines for adapting cognitive stimulation therapy to other cultures. Clin Interv Aging. (2014) 9:1003–7. 10.2147/CIA.S61849 25061282 PMC4079629

[26] Paroutis S Heracleous L Angwin D Practicing Strategy: Text and Cases. London, Sage (2016).

[27] Stoner CR Lakshminarayanan M Durgante H Spector A. Psychosocial interventions for dementia in low- and middle-income countries (LMICs): a systematic review of effectiveness and implementation readiness. Aging & Mental Health. (2019) 1–12. 10.1080/13607863.2019.1695742. [Epub ahead of print]. 31814427 PMC8026009

[28] Shayo EH Senkoro KP Momburi R Olsen ØE Byskov J Makundi EA. Access and utilisation of healthcare services in rural Tanzania: a comparison of public and non-public facilities using quality, equity, and trust dimensions. Global Public Health. (2016) 11:407–22. 10.1080/17441692.2015.1132750 26883021

[29] Prince MJ. The 10/66 dementia research group - 10 years on. Ind J Psych. (2009) 51:S8–S15. 21416024 PMC3038536

[30] Kirk MA Kelley C Yankey N Birken SA Abadie B Damschroder L A systematic review of the use of the Consolidated Framework for Implementation Research. Implem Sci. (2016) 11:72 10.1186/s13012-016-0437-z PMC4869309 27189233

