# Supplementary material for: A New Approach for Developing “Implementation Plans” for Cognitive Stimulation Therapy (CST) in Low and Middle-Income Countries: Results From the CST-International Study
Source: Front Public Health. 2020 Jul 31;8:342. doi: 10.3389/fpubh.2020.00342 (PMC7411173; doi:10.3389/fpubh.2020.00342)
Supplement: Supplementary file 1 [file Data_Sheet_1.PDF]

CST-International
BRAZIL, INDIA & TANZANIA

Cognitive Stimulation Therapy (CST) for People with Dementia: Implementation Plan for Rio de Janeiro and São Paulo, Brazil.

Table of Contents

Table of Contents **2**

Guide to Using the Implementation and Action Plans **4**

Summary of Responses and Justification for Selected Mechanisms **4**

Responder Information 4

Justification for Selected Mechanisms 5

Implementation Plan **7**

Travel 7

Knowledge/ beliefs about the intervention 7

Relative Advantage 8

Costs (Direct and Indirect) 8

Adaptability 8

Networks and Communications 9

External policy and incentives 10

Structural Characteristics 10

Individual Stage of Changes 11

Action Plans **12**

Travel 12

Knowledge/ Beliefs about the Intervention 12

Relative Advantage 12

Costs (Direct and Indirect) 13

Adaptability 13

Network and Communications 13

External policy and incentives 14

Structural Characteristics 14

Individual Stage of Changes 15

Action Plans for Individual Investigators 16

Researcher 1 16

Researcher 2 17

Researcher 3 18

Researcher 4 18

Researcher 5 20

Researcher 6 21

Researcher 7 22

Researcher 8 22

Researcher 9 23

Researcher 10 23

Researcher 11 23

Individual CST Facilitators 23

Appendix 1: Barriers and Facilitators Local Checklist 25

Guide to Using the Implementation and Action Plans

Headings for each section refer to categories from the Consolidated Framework for Implementation Research (CFIR), which were identified as part of stakeholder meetings in Tanzania. The CFIR underpins and informs all work conducted as part of CST-International. Headings are then further divided into mechanisms that are 'essential' and mechanisms that researchers can 'further consider'. Mechanisms that are essential will be implemented by CST-International investigators in Brazil and 'further consider' mechanisms can be implemented if the team feel there is sufficient resources available. Each mechanism selected for use has been justified (pg 5 – 6). This is followed by a written summary of the mechanisms, with hyperlinks to specific 'action plans' associated with each of the categories (pg 12 – 15). Researchers can refer to these action plans and use them to monitor progress. For ease, the last section of this plan (pg 16 – 24) contains all of the aforementioned action plans but compiled for each individual investigator. An effort has been made to divide these equally amongst staff, allowing for time allocated to the grant (e.g. 5% FTE versus 10% FTE). Finally, the local barrier and facilitator checklist is included as Appendix 1 (pg 25).

Summary of Responses and Justification for Selected Mechanisms

Responder Information

Thirty-three stakeholders, identified during Phase One of the trial, rated how essential and easy the identified mechanism to support implementation in Brazil were to use. In brief, responders were asked to rate each of the 41 identified mechanisms, according to the below matrix and the modes for each of the mechanisms were examined.

The mechanism is:

- **1** = Advisory and Difficult (AD)
- **2** = Advisory and Intermediate (AI)
- **3** = Advisory and Easy (AE)
- **4** = Desirable and Difficult (DD)
- **5** = Essential and Difficult (ED)
- **6** = Desirable and Intermediate (DI)
- **7** = Desirable and Easy (DE)
- **8** = Essential and Intermediate (EI)
- **9** = Essential and Easy (EE)

For the full methodology associated with this stage, please see the documents entitled ‘Stakeholder Methods V2’ and ‘Implementation Plan Methods V2’.

Responders consisted of five members of the CST-International research team (two psychologists, one psychiatrist, one PhD candidate and one research assistant) and sixteen potential CST facilitators (Group 2), for whom professions included psychologists, nurses, physicians, gerontologist, speech therapist, recreation worker and social worker. Six decision makers from the private and public sectors responded on behalf of Group 1 (local policy or decision makers). For Group 3 (people with dementia, caregivers and other interested parties), six caregivers responded. In addition to rating the table, 4 people gave additional qualitative feedback.

Justification for Selected Mechanisms

The most common individual rating for mechanisms was ‘8’ (Essential and Intermediate), however, there was a large amount of variance in responses. For example, the mechanism ‘CST training should be delivered where CST sessions are going to be offered’ was rated as advisory and intermediate (2) by two responders and essential and easy (9) by two responders. All mechanisms were discussed at a local team meeting, with reference to how many could be implemented and the prioritisation of those mechanisms that were rated as both ‘essential’ for implementation and ‘easy’ to use. It was decided that mechanisms rated as essential and easy/intermediate to implement would always be categorised as ‘essential’ and mechanisms with lower modes would be categorised as ‘further consider. A draft implementation plan was then circulated, and CST-Investigators were invited to comment and refine the plan, before a finalised plan was agreed upon.

Travel

Two desirable mechanisms for travel were identified, both related to settings where CST should be offered in order to reduce patients’ travel time and will be further considered.

Knowledge/ beliefs about the intervention

Seven essential mechanisms were identified. They were all related to training courses and will be used.

Cost (direct and indirect)

Costs are likely to have a significant impact on the implementation of CST and should be prioritised. One mechanism was identified as essential and two were identified as desirable but it was also identified as being easy to implement, so both mechanisms will be used.

Adaptability

Three mechanisms were deemed essential and of intermediate difficulty to use. All mechanisms relate to creating new material for CST in order to adapt the therapy for different populations (e.g. illiterate people). These mechanisms will be considered after the data collection phase.

Relative advantage

Two mechanisms were rated as essential and easy. One was rated desirable and intermediate, but as it relates to the training course, it can be incorporated into similar actions from previous implementation areas, with no additional resources needed. All mechanisms for this stage will be used.

Network and Communications

Three mechanisms were rated as essential and easy and three as essential and intermediate. All mechanisms were related to advertising CST and will be used.

External policy and incentives

One mechanism was rated as essential and intermediate, one as essential and difficult and one as advisory and difficult. The mechanism rated as essential and intermediate will be used, there others will be further considered.

Structural characteristics

Nine mechanisms were rated as essential and easy or intermediate, four were related to available time for facilitators to be trained and to run CST groups, two were related to the changes in the service organization in order to include CST sessions, one was related to appropriate meeting space, one was related to available time for PwD and carers to attend

CST sessions and one was related to general instruction about CST for staff, all nine mechanisms will be used. Also two mechanisms were rated as desirable and intermediate and one as essential but difficult to implement, these mechanisms will be further considered.

Individual Stage of Changes

One mechanism was rated as desirable and intermediate and related to training for carers regarding individual CST. It will be further considered.

Implementation Plan

Travel

Further consider

1) *CST Sessions in local community settings:* In order to facilitate patients' access to CST, CST-Investigators should approach leaders in community settings as possible sites offering CST (action 1.1).
2) *CST Sessions in hospital outpatient clinics:* In order to facilitate patients' access to CST, CST-Investigators should approach managers of hospital outpatient clinics as possible sites offering CST (action 1.2).

Knowledge/ beliefs about the intervention

Essential

1) *Development of a Dementia Awareness Course (DAC):* This will be primarily aimed at educating carers of people with dementia. The three-hour course will contain information regarding the difference between natural aging and dementia, designed to combat the stigma of dementia, as well as information on other pharmacological and non-pharmacological treatments for people with dementia (action 2.1). Cultural adaptations for the Brazilian population, with specific signposting for each site, will be made by Researcher 1 and Researcher 8, in Rio de Janeiro and São José dos Campos, respectively (action 2.2). The course material will be translated Researcher 3 (action 2.3). Attendees will be invited by Researcher 3 (Rio de Janeiro) and by Researcher 6 (São José dos Campos) (action 2.4). The course will be run by Researcher 1 in Rio de Janeiro and Researcher 6 in São José dos Campos (actions 2.5).

2) *Training for CST-facilitators*: Considering that dementia is not included in formal curriculum for healthcare professionals' formation, potential CST-facilitators should attend the DAC as part of the CST training (action 2.6).

Relative Advantage

Essential

1) *Use evidence-based benefits of CST to recruit sites:* As a strategy to recruit sites, CST-Investigators will explain the evidence-based benefits to sites managers when approaching/recruiting sites (action 3.1).
2) *Explain benefits of CST to people with dementia and their caregivers*: Benefits of CST will be explained to people with dementia and their caregivers in order to increase patients and caregivers' adherence. It will be incorporated into the DAC (actions 2.7 and 2.8) delivered as part of the intervention.
3) *Explain benefits of CST to CST-facilitators:* During CST training, benefits of CST will be explained to possible CST-facilitators in order to help recruitment of CST-facilitators and increase motivation to deliver CST sessions (action 3.2).

Costs (Direct and Indirect)

Essential

1) *CST training delivered where CST session are going to be offered:* To avoid additional costs for future sites and CST-facilitators, CST-Investigators will organize CST training on sites where CST sessions are going to be offered (action 4.1).
4) *Provide CST cost-effectiveness analysis:* CST-Investigators should discuss the cost-effectiveness analysis indicating CST is a cheaper alternative with sites managers when approaching/recruiting sites. It will be incorporated into the strategies to recruit sites (action 3.1).
2) *Utilize already available resources*: In order to limit the cost of the intervention, CST-facilitators should utilize the resources already available on site as material for the CST sessions (action 4.2).

Adaptability

Further consider

1) *Adapt CST material to illiterate people, people with disabilities and people with severe dementia*: As illiteracy is common in the Brazilian population, CST-Investigators should develop CST material for this specific population (action 5.1). In order to include all people with dementia, CST-Investigators should also consider developing CST material for people with disabilities and people with severe dementia (actions 5.2 and 5.3).

Networks and Communications

Essential

1) *Advertising CST groups at carer associations:* CST-Investigators will contact carer associations to facilitate advertising CST groups at national and regional levels. This will include information on CST and where people with dementia and their carers can access the therapy or further information (action 6.1). Advertising strategies should include non-stigmatizing words.
2) *Advertising CST groups at geriatrician associations:* CST-Investigators will contact geriatrician associations to facilitate advertising CST groups at national and regional levels. This will include information on CST and where people with dementia and their carers can access the therapy or further information (action 6.2). Advertising strategies should include non-stigmatizing words.
3) *Local advertising:* CST will be advertised through community centres, specialized polyclinics and via general practitioners. Investigators should attempt to ensure that costs are minimal and that it is an effective use of time. Advertising must include information on CST and where people with dementia and their carers can access the therapy or further information (action 6.3). Advertising strategies should include non-stigmatizing words.
4) *Identify key spokesperson*: A key spokesperson will be identified in each site (carer associations, geriatrician associations, community centres, etc), in order to facilitate advertising CST groups to people with dementia and their carers (action 6.4).

Whilst not discussed by groups, some aspects of network and communication are a requirement of CST implementation and will therefore be included in the present implementation plan.

5) *Development of a Master Training Programme:* A training course for 'master trainers' will be developed in the UK, primarily by Researcher 10 (action 6.5). This will be used to increase the pool of available trainers in all countries.
6) *Supervision of CST Facilitators:* Newly trained CST facilitators will have regular supervision, using the most appropriate means available (e.g. WhatsApp if necessary or face to face) (action 6.6).

External policy and incentives

Essential

1) *Liaison with government stakeholders:* CST-Investigators should contact government stakeholders to discuss the possibility of implement CST at a policy level (action 7.1).

Further consider

2) *Public and Private funding:* CST-Investigators should contact people responsible for decision making at a policy level in public and private domains to discuss the creation of private and public funding mechanisms for implementation of CST (action 7.2).
3) *Insurance Coverage*: Investigators should consider contacting prominent insurance companies to determine whether CST would be covered by insurers as part of a 'talking therapy' (action 7.3)

Structural Characteristics

Essential

1) *Protected time:* CST facilitators will need protected time allocated for CST (training and running sessions), as well for updating their knowledge about dementia in general. CST-Investigators should inform site management that protected time for staff is a requirement of the project when approaching/ recruiting sites and ensure that site managers agree to this (action 8.1).
2) *Changes in site organization:* CST-Investigators should inform site management that inclusion of CST into the weekly routine is a requirement of the project when approaching/ recruiting sites and ensure that site managers agree to this (action 8.2).
3) *Planning CST training:* CST training will be offered on different days/times in order to attend the needs of potential CST-facilitators (action 8.3).

4) *Planning CST sessions*: Considering that CST is a 7-week programme and that two CST-facilitators are needed, CST-facilitators will plan the sessions according the staff holiday schedule (action 8.4). In order to increase adherence CST-facilitators will agree with carers which days and times are best for running CST groups (action 8.5).
5) *Minimal availability of space*: When approaching/ recruiting sites, CST-Investigators will check that the site has an available space appropriate for running CST sessions (action 8.6).
6) *Information regarding CST for staff*: The staff of the site where CST is being delivered will receive information regarding CST and instruction on how to help facilitate the implementation (e.g. avoid interrupting the session) (action 8.7).

Further consider

7) *Combination of CST Sessions:* Sessions can be combined, or not, depending on the context (e.g. in long term care facilities where patients are living on site combination of sessions should be avoided). CST facilitators should agree with carers whether sessions should run once or twice a week (action 8.8).
8) *Access for people with disabilities*: When approaching/recruiting sites, CST-Investigators should prefer sites that can be easily accessed by people with disabilities (action 8.9).
9) *Identification of underdiagnosed cases of dementia*: CST-Investigators should meet with teams in charge for screening for dementia in each site to identify potential underdiagnosed cases (action 8.10).

Individual Stage of Changes

Further consider

1) *Training for carers to deliver individual Cognitive Stimulation Therapy (iCST):* Carers should be trained to deliver iCST to patient after the end of the CST sessions (action 9.1).

Action Plans

Travel

Further consider

Site: Rio de Janeiro / São José dos Campos Country: Brazil		
**Action to be undertaken**	**Team member responsible for**	**Due by**
1.1 CST-Investigators should approach leaders in community settings as possible sites offering CST	Researchers 2, 4 and 5	31st July (2020)
1.2 CST-Investigators should approach managers of hospital outpatient clinics as possible sites offering CST	Researchers 2, 4 and 5	31st July (2020)

Knowledge/ Beliefs about the Intervention

Essential

Site: Rio de Janeiro / São José dos Campos Country: Brazil		
**Action to be undertaken**	**Team member responsible for**	**Due by**
2.1 Develop a 3-hour dementia awareness course for family carers, with information on stigma, pharmacological and non-pharmacological interventions included	Researcher 11	31st May (2019)
2.2 Cultural adaptations of the Dementia Awareness Courses (DAC) with specific signposting for each site	Researchers 1 and 8	31st August (2019)
2.3 Translation of the DAC material into Portuguese	Researcher 3	30th September (2019)
2.4 Invite and organise (room booking etc) DAC for family carers and possible CST-facilitators	Researcher 3 and 6	30th June (2020)
2.5 Deliver the three hour DAC at regular intervals	Researchers 1 and 6	30th June (2020)

Relative Advantage

Essential

Site: Rio de Janeiro / São José dos Campos Country: Brazil		
**Action to be undertaken**	**Team member responsible for**	**Due by**

3.1 CST-Investigators will explain the evidence-based benefits and the cost-effectiveness analysis indicating CST is a cheaper alternative to sites managers when approaching/recruiting sites	Researchers 2, 4 and 5	31st July (2020)
3.2 CST-Investigators will explain the benefits of CST to possible CST-facilitators	Researchers 1 and 6	31st July (2020)

Costs (Direct and Indirect)

Essential

**Site: Rio de Janeiro / São José dos Campos Country: Brazil**		
**Action to be undertaken**	**Team member responsible for**	**Due by**
4.1 CST-Investigators will organize CST training on sites where CST sessions are going to be offered	Researchers 1 and 6	31st July (2020)
4.2 CST-facilitators should utilize the resources already available on site as material for the CST sessions	Individual CST Facilitators	31st July (2020)

Adaptability

Further consider

**Site: Rio de Janeiro / São José dos Campos Country: Brazil**		
**Action to be undertaken**	**Team member responsible for**	**Due by**
5.1 Adapt CST material to illiterate people, people with disabilities and people with severe dementia	All Researchers	31st August (2021)

Network and Communications

Essential

**Site: Rio de Janeiro / São José dos Campos Country: Brazil**		
**Action to be undertaken**	**Team member responsible for**	**Due by**
6.1 CST-Investigators will contact carer associations to facilitate advertising CST groups at national and regional levels	Researchers 1 and 6	30th June (2020)

6.2 CST-Investigators will contact geriatrician associations to facilitate advertising CST groups at national and regional levels	Researchers 5 and 9	30th June (2020)
6.3 CST will be advertised through community centres, specialized polyclinics and via general practitioners	Researchers 1 and 6	30th June (2020)
6.4 A 'master trainer' course for CST facilitators will be developed to increase the pool of trainers in Brazil	Researcher 10	30th June (2020)
6.5 Offer regular supervision to newly trained CST facilitators	Researchers 1 and 3	31st August (2021)

External policy and incentives

Essential

**Site: Rio de Janeiro / São José dos Campos Country: Brazil**		
**Action to be undertaken**	**Team member responsible for**	**Due by**
7.1 Contact with government stakeholders to discuss the possibility of implement CST at a policy level	Researchers 2, 4 and 5	31st August (2021)

Further consider

**Site: Rio de Janeiro / São José dos Campos Country: Brazil**		
**Action to be undertaken**	**Team member responsible for**	**Due by**
7.2 Contact with people responsible for decision making at a policy level in public and private domains to discuss the creation of private and public funding mechanisms for implementation of CST	Researchers 4 and 5	31st August (2021)
7.3 Contact with prominent insurance companies to determine whether CST would be covered by insurers as part of a 'talking therapy'	Researcher 4	31st August (2021)

Structural Characteristics

Essential

**Site: Rio de Janeiro / São José dos Campos Country: Brazil**

Action to be undertaken	Team member responsible for	Due by
8.1 Inform site management of protected time requirement for CST facilitators and ensure permission is granted	Researchers 1 and 6	30th June (2020)
8.2. Inform site management that inclusion of CST into the weekly routine is a requirement	Researchers 1 and 6	30th June (2020)
8.3 Offer CST training on different days/times	Researchers 1 and 6	30th June (2020)
8.4 CST-facilitators will plan the sessions according the staff holiday schedule	Individual CST Facilitators	30th June (2020)
8.5 CST-facilitators will agree with carers which days and times are best for running CST groups	Individual CST Facilitators	30th June (2020)
8.6 Check that the site has an available space appropriate for running CST sessions	Researchers 1 and 6	30th June (2020)
8.7 Offer information regarding CST to staff of the site where CST is being delivered	Researchers 1 and 6	30th June (2020)

Further consider

**Site: Rio de Janeiro / São José dos Campos Country: Brazil**		
**Action to be undertaken**	**Team member responsible for**	**Due by**
8.8 CST facilitators should agree with carers how often their CST group should run (one or twice weekly)	Individual CST Facilitators	30th June (2020)
8.9 CST-Investigators should prefer sites that can be easily accessed by people with disabilities	Researchers 1 and 6	30th June (2020)
8.10 CST-Investigators should meet with teams in charge for screening for dementia in each site to identify potential underdiagnosed cases	Researchers 1 and 6	30th June (2020)

Individual Stage of Changes

Further consider

**Site: Rio de Janeiro / São José dos Campos Country: Brazil**		
**Action to be undertaken**	**Team member responsible for**	**Due by**

9.1 Contact with government stakeholders to discuss the possibility of implement CST at a policy level	Researchers 4 and 5	31st August (2021)

Action Plans for Individual Investigators

Researcher 1

**CFIR Category**	**Action Point**	**Due by**	**Completed on**
	**ESSENTIAL**		
Knowledge/ Beliefs about the Intervention	Cultural adaptations of the Dementia Awareness Courses (DAC) with specific signposting for each site	31.08.2019	20.08.2019
Knowledge/ Beliefs about the Intervention	Deliver the three hour DAC at regular intervals	30.06.2020	
Relative Advantage	CST-Investigators will explain the benefits of CST to possible CST-facilitators	31.07.2020	
Costs	CST-Investigators will organize CST training on sites where CST sessions are going to be offered	31.07.2020	
Network and Communications	CST-Investigators will contact carer associations to facilitate advertising CST groups at national and regional levels	30.06.2020	
Network and Communications	CST will be advertised through community centres, specialized polyclinics and via general practitioners	30.06.2020	
Network and Communications	Offer regular supervision to newly trained CST facilitators	31.08.2021	

Structural Characteristics	Inform site management of protected time requirement for CST facilitators and ensure permission is granted	30.06.2020	
Structural Characteristics	Inform site management that inclusion of CST into the weekly routine is a requirement	30.06.2020	
Structural Characteristics	Offer CST training on different days/times	30.06.2020	
Structural Characteristics	Check that the site has an available space appropriate for running CST sessions	30.06.2020	
Structural Characteristics	Offer information regarding CST to staff of the site where CST is being delivered	30.06.2020	
**FURTHER CONSIDER**			
Adaptability	Adapt CST material to illiterate people, people with disabilities and people with severe dementia	31.08.2021	
Structural Characteristics	CST-Investigators should prefer sites that can be easily accessed by people with disabilities	30.06.2020	
Structural Characteristics	CST-Investigators should meet with teams in charge for screening for dementia in each site to identify potential underdiagnosed cases	30.06.2020	

Researcher 2

CFIR Category	Action Point	Due by	Completed on
**ESSENTIAL**			
Travel	CST-Investigators should approach leaders in community settings as possible sites offering CST	31.07.2020	
Travel	CST-Investigators should approach managers of hospital outpatient clinics as possible sites offering CST	31.07.2020	

Relative Advantage	CST-Investigators will explain the evidence-based benefits and the cost-effectiveness analysis indicating CST is a cheaper alternative to sites managers when approaching/recruiting sites	31.07.2020	
External policy and incentives	Contact with government stakeholders to discuss the possibility of implement CST at a policy level	31.08.2021	
**FURTHER CONSIDER**			
Adaptability	Adapt CST material to illiterate people, people with disabilities and people with severe dementia	31.08.2021	

Researcher 3

CFIR Category	Action Point	Due by	Completed on
**ESSENTIAL**			
Knowledge/ Beliefs about the Intervention	Translation of the DAC material into Portuguese	30.09.2019	27.09.2019
Knowledge/ Beliefs about the Intervention	Invite and organise (room booking etc) DAC for family carers and possible CST-facilitators	30.06.2020	
Network and Communications	Offer regular supervision to newly trained CST facilitators	31.08.2021	
**FURTHER CONSIDER**			
Adaptability	Adapt CST material to illiterate people, people with disabilities and people with severe dementia	31.08.2021	

Researcher 4

CFIR Category	Action Point	Due by	Completed on

ESSENTIAL			
Travel	CST-Investigators should approach leaders in community settings as possible sites offering CST	31.07.2020	
Travel	CST-Investigators should approach managers of hospital outpatient clinics as possible sites offering CST	31.07.2020	
Relative Advantage	CST-Investigators will explain the evidence-based benefits and the cost-effectiveness analysis indicating CST is a cheaper alternative to sites managers when approaching/recruiting sites	31.07.2020	
External policy and incentives	Contact with government stakeholders to discuss the possibility of implement CST at a policy level	31.08.2021	
**FURTHER CONSIDER**			
Adaptability	Adapt CST material to illiterate people, people with disabilities and people with severe dementia	31.08.2021	
External policy and incentives	Contact with people responsible for decision making at a policy level in public and private domains to discuss the creation of private and public funding mechanisms for implementation of CST	31.08.2021	
External policy and incentives	Contact with prominent insurance companies to determine whether CST would be covered by insurers as part of a ‘talking therapy’	31.08.2021	
Individual Stage of Changes	Contact with government stakeholders to discuss the possibility of implement CST at a policy level	31.08.2021	

Researcher 5

CFIR Category	Action Point	Due by	Completed on
**ESSENTIAL**			
Network and Communications	CST-Investigators will contact geriatrician associations to facilitate advertising CST groups at national and regional levels	30.06.2020	
Travel	CST-Investigators should approach leaders in community settings as possible sites offering CST	31.07.2020	
Travel	CST-Investigators should approach managers of hospital outpatient clinics as possible sites offering CST	31.07.2020	
Relative Advantage	CST-Investigators will explain the evidence-based benefits and the cost-effectiveness analysis indicating CST is a cheaper alternative to sites managers when approaching/recruiting sites	31.07.2020	
External policy and incentives	Contact with government stakeholders to discuss the possibility of implement CST at a policy level	31.08.2021	
**FURTHER CONSIDER**			
Adaptability	Adapt CST material to illiterate people, people with disabilities and people with severe dementia	31.08.2021	
External policy and incentives	Contact with people responsible for decision making at a policy level in public and private domains to discuss the creation of private and public funding mechanisms for implementation of CST	31.08.2021	
Individual Stage of Changes	Contact with government stakeholders to discuss the possibility of implement CST at a policy level	31.08.2021	

Researcher 6

CFIR Category	Action Point	Due by	Completed on
**ESSENTIAL**			
Knowledge/ Beliefs about the Intervention	Deliver the three hour DAC at regular intervals	30.06.2020	
Relative Advantage	CST-Investigators will explain the benefits of CST to possible CST-facilitators	31.07.2020	
Costs (Direct and Indirect)	CST-Investigators will organize CST training on sites where CST sessions are going to be offered	31.07.2020	
Network and Communications	CST-Investigators will contact carer associations to facilitate advertising CST groups at national and regional levels	30.06.2020	
Network and Communications	CST will be advertised through community centres, specialized polyclinics and via general practitioners	30.06.2020	
Structural Characteristics	Inform site management of protected time requirement for CST facilitators and ensure permission is granted	30.06.2020	
Structural Characteristics	Inform site management that inclusion of CST into the weekly routine is a requirement	30.06.2020	
Structural Characteristics	Offer CST training on different days/times	30.06.2020	
Structural Characteristics	Check that the site has an available space appropriate for running CST sessions	30.06.2020	
Structural Characteristics	Offer information regarding CST to staff of the site where CST is being delivered	30.06.2020	
**FURTHER CONSIDER**			

Adaptability	Adapt CST material to illiterate people, people with disabilities and people with severe dementia	31.08.2021	
Structural Characteristics	CST-Investigators should prefer sites that can be easily accessed by people with disabilities	30.06.2020	
Structural Characteristics	CST-Investigators should meet with teams in charge for screening for dementia in each site to identify potential underdiagnosed cases	30.06.2020	

Researcher 7

CFIR Category	Action Point	Due by	Completed on
**ESSENTIAL**			
Knowledge/ Beliefs about the Intervention	Invite and organise (room booking etc) DAC for family carers and possible CST-facilitators	30.06.2020	
**FURTHER CONSIDER**			
Adaptability	Adapt CST material to illiterate people, people with disabilities and people with severe dementia	31.08.2021	

Researcher 8

CFIR Category	Action Point	Due by	Completed on
**ESSENTIAL**			
Knowledge/ Beliefs about the Intervention	Cultural adaptations of the Dementia Awareness Courses (DAC) with specific signposting for each site	31.08.2019	20.08.2019
**FURTHER CONSIDER**			

Adaptability	Adapt CST material to illiterate people, people with disabilities and people with severe dementia	31.08.2021	

Researcher 9

**CFIR Category**	**Action Point**	**Due by**	**Completed on**
**ESSENTIAL**			
Network and Communications	CST-Investigators will contact geriatrician associations to facilitate advertising CST groups at national and regional levels	30.06.2020	

Researcher 10

**CFIR Category**	**Action Point**	**Due by**	**Completed on**
**ESSENTIAL**			
Network and Communications	A 'master trainer' course for CST facilitators will be developed to increase the pool of trainers in Brazil	30.06.2020	

Researcher 11

**CFIR Category**	**Action Point**	**Due by**	**Completed on**
**ESSENTIAL**			
Knowledge/ Beliefs about the Intervention	Develop a 3-hour dementia awareness course for family carers, with information on stigma, pharmacological and non-pharmacological interventions included	31.05.2019	

Individual CST Facilitators

**CFIR Category**	**Action Point**	**Due by**	**Completed on**
**ESSENTIAL**			

Costs (Direct and Indirect)	CST-facilitators should utilize the resources already available on site as material for the CST sessions	31.07.2020	
Structural Characteristics	CST-facilitators will plan the sessions according the staff holiday schedule	30.06.2020	
Structural Characteristics	CST-facilitators will agree with carers which days and times are best for running CST groups	30.06.2020	
**FURTHER CONSIDER**			
Structural Characteristics	CST facilitators should agree with carers how often their CST group should run (one or twice weekly)	30.06.2020	

Appendix 1: Barriers and Facilitators Local Checklist

Local Checklist

Below are some commonly encountered problems when running CST groups. This checklist should be completed by a CST facilitator prior to running their first group.

→ Location of Group (building and region)

____

→ Participants

- ☐ Do all of your participants meet the ICD-10 criteria for dementia?

→ Facilities

- ☐ Will the session be held in a private place where participants will not be interrupted?
- ☐ Is the CST session in a neutral setting (e.g. it is not in a church)?
- ☐ Will there be access to drinking water?
- ☐ Is there a toilet nearby?
- ☐ Is there a table that can be used?
- ☐ Are there enough chairs?
- ☐ Are the chairs the right height?
- ☐ Is the place accessible for those with reduced mobility (e.g. wheelchair)?

→ Travel and Timing

- ☐ Can the building be reached by public transport?
- ☐ Is the building close enough to people's homes?
- ☐ Have you agreed which times and dates the CST sessions will be with carers?
- ☐ Have you checked if any holidays will interfere with the continuity of the sessions?

→ Materials

- ☐ Have you got all the materials you need (e.g. local foods for the food session)?
- ☐ Have you got all the electronic devices needed for the sessions? (e.g. notebook or cell phone with a playlist with the songs used in the session)?
