# Supplementary material for: A New Approach for Developing “Implementation Plans” for Cognitive Stimulation Therapy (CST) in Low and Middle-Income Countries: Results From the CST-International Study
Source: Front Public Health. 2020 Jul 31;8:342. doi: 10.3389/fpubh.2020.00342 (PMC7411173; doi:10.3389/fpubh.2020.00342)
Supplement: Supplementary file 2 [file Data_Sheet_2.PDF]

CST-International
BRAZIL, INDIA & TANZANIA

Phase 1 Stakeholder Questions

Essential Questions

These questions should be put to stakeholders in each group.

Group 1	*Group 2*	*Group 3*
1) How important/ necessary is it to implement CST? What has influenced this answer?<br>*(Implementation climate)*<br><br>2) What (national) guidelines regarding dementia treatments are available and what guidelines should CST be in at the end of the study?<br>*External policies and incentives*<br><br>3) What investment is needed for CST to be implemented effectively?<br>*(cost)*<br><br>4) What support is available to local teams to help them implement CST?<br>*(Implementation climate)*<br><br>5) Would you integrate CST into national programmes? What has influenced this decision<br>*(evidence strength)*	1) What are the known barriers people encounter when accessing services?<br>*(patient needs and resources)*<br><br>2) What training/ support will staff need to implement CST e.g. time off regular duties/ travel for training?<br>*(implementation climate)*<br><br>3) CST runs for 7 consecutive weeks. Are there enough staff available who could commit to the whole programme?<br>*(structural characteristics)*<br><br>4) What are the particular challenges that CST implementation would face in your service e.g. travel/room space?<br>*(complexity)*<br><br>5) Would you implement CST in your practice? What aspects of CST have influenced this decision?<br>*(Implementation climate)*	1) What can you tell me about dementia/ memory problems?<br>*(culture)*<br><br>2) What are the effects of CST?<br>*(evidence strength and quality)*<br><br>3) If CST was available at your local heath care facility, would you/ would your family member with dementia participate? What aspects of CST have influenced this decision<br>*(patient needs and resources)*<br><br>4) What would make you more likely to attend CST?<br>*(culture)*<br><br>5) What would make you less likely to attend CST?<br>*(culture)*

<u>Supplementary Questions</u>

These questions can be asked after answers to essential questions have been received. Facilitators do not need to ask every question but should choose the most appropriate questions and can adapt the wording as they wish.

Group 1	***Group 2***	***Group 3***
1) What can you tell me about dementia? *(culture)*	1) How can dementia be treated? *(culture)*	1) How can dementia/ memory problems be treated? *(culture)*
2) How can dementia be treated? *(culture)*	2) What healthcare treatments are available to people with dementia? *(patient needs and resources)*	2) What healthcare treatments are available to people with dementia or memory problems? *(patient needs and resources)*
3) What healthcare treatments are available to people with dementia? *(patient needs and resources)*	3) How important/ necessary is it to implement CST? (*implementation climate)*	3) What stops people getting treatment for illnesses/ diseases? *(patient needs and resources)*
4) What are the known barriers people encounter when accessing services? *(patient needs and resources)*	4) How should CST be changed to ensure it is tailored to this country? *(adaptability)*	4) In what way do you think CST could benefit you? *(culture)*
5) What are the advantages of implementing CST? *(relative advantage)*	5) How will local needs influence how CST is used? *(adaptability)*	
6) What are the disadvantages of implementing CST? *(relative advantage)*	6) What adaptions will need to be made to the CST manual? *(adaptability)*	
7) What organisations/ charities/ government bodies are available to you to help support implementation? *(cosmopolitanism)*	7) What changes will need to be made to the one-day training model? *(adaptability)*	

8) Are there any government policies that may encourage implementation? *(external policies and incentives)*<br><br>9) How are decisions about dementia treatments made (e.g. centralised/ localised)? *(structural characteristics)*<br><br>10) Are you aware of other countries/ organisations implementing CST? How has this affected your views on implementing it? *(peer pressure)*	8) How will the implementation of CST affect normal activities/ practices? *(complexity)*<br><br>9) What organisations/ charities/ government bodies are available to you to help support implementation? *(cosmopolitanism)*<br><br>10) Are you aware of other countries/ organisations implementing CST? How has this affected your views on implementing it? *(peer pressure)*	
